# A lost race against time—a rare case of AL amyloidosis

**DOI:** 10.1007/s40477-025-01020-z

**Published:** 2025-04-25

**Authors:** Inês Conde, Mónica Dias, Sofia Fernandes, Nuno Antunes, Rui Flores, Carlos Galvão Braga

**Affiliations:** Cardiology Department, Hospital de Braga, São Victor, 4710-243 Braga, Portugal

**Keywords:** Cardiac amyloidosis, Light-chain amyloidosis, Endomyocardial biopsy

## Abstract

**Introduction:**

Amyloidosis is a rare multi-system disease that can affect the heart. Recent advances have been made in finding successful treatments for these patients. Nonetheless, cardiac amyloidosis continues to carry high morbidity and mortality.

**Case presentation:**

This case refers to a 52-year-old man presenting with signs and symptoms of heart failure. Electrocardiogram showed q waves in V1-V3 and T wave inversion in the inferior and lateral leads. Echocardiography revealed moderate concentric left ventricular hypertrophy, with preserved biventricular systolic function and no other changes. Cardiac magnetic resonance showed severe left ventricular hypertrophy and diffuse fibrosis, suggesting infiltrative cardiomyopathy. Given these findings, a diagnosis of amyloidosis was considered and complementary tests were requested. Laboratory tests showed elevation of Kappa light chains in urine, with normal serum light chains. Cardiac scintigraphy with Tec99m-DPD showed increased cardiac uptake. Genetic testing showed no clinically relevant variants in the TTR gene. Salivary gland and abdominal fat biopsy demonstrated no deposition of amyloid substance. Bone marrow evaluation demonstrated the presence of 0.8% plasma cells (99.2% clonal). Given that a high degree of suspicion remained, an endocardial biopsy was performed and immunohistochemical staining revealed deposits of kappa light chains, suggesting AL amyloidosis. However, in the course of the clinical investigation, the patient's condition progressively deteriorated. By the time AL amyloidosis was diagnosed and targeted therapy initiated, the patient was already severely debilitated, ultimately leading to a short-term fatal outcome.

**Comment:**

Amyloidosis, often undiagnosed early, can severely affect multiple organs, particularly the heart, leading to poor prognosis without timely intervention. In this case, AL amyloidosis, a type where plasma cells produce amyloidogenic light chains, was identified. The patient’s late diagnosis led to rapid progression and treatment with chemotherapy and steroids; however, his condition deteriorated quickly, resulting in a fatal outcome.

## Introduction

Amyloidosis, a rare multisystemic disease, may impact the heart with high morbidity and mortality. This case highlights delayed diagnosis in a patient with cardiac amyloidosis due to the absence of multiple myeloma diagnostic criteria, emphasizing the need for recognizing other infiltrating heart diseases.

## Case presentation

**Fig. 1:**
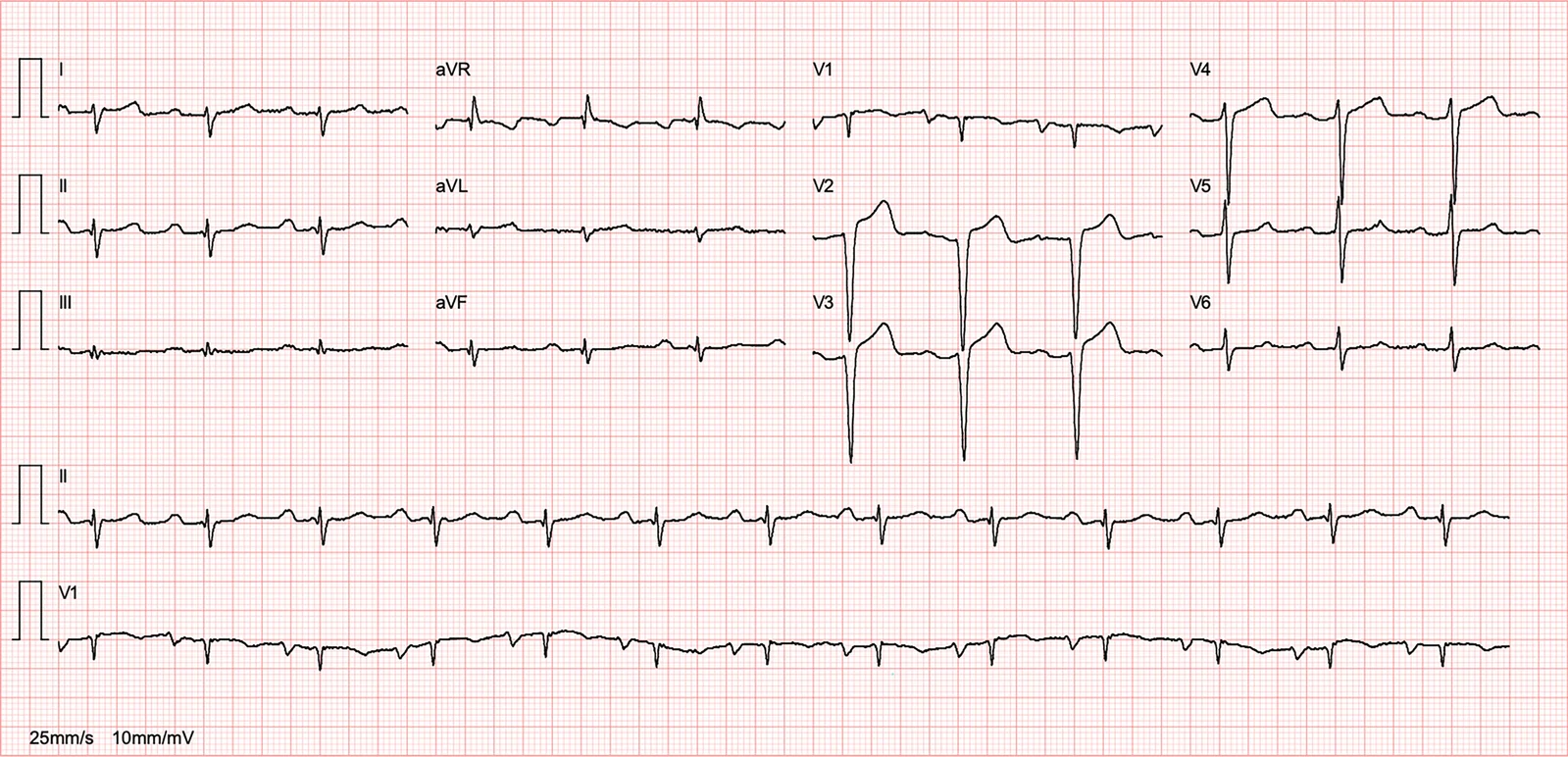
Electrocardiogram, showing sinus rhythm, first-degree atrioventricular block, Q-waves in V1-V3 precordial leads, and T-wave inversion in the inferior and lateral leads

**Fig. 2:**
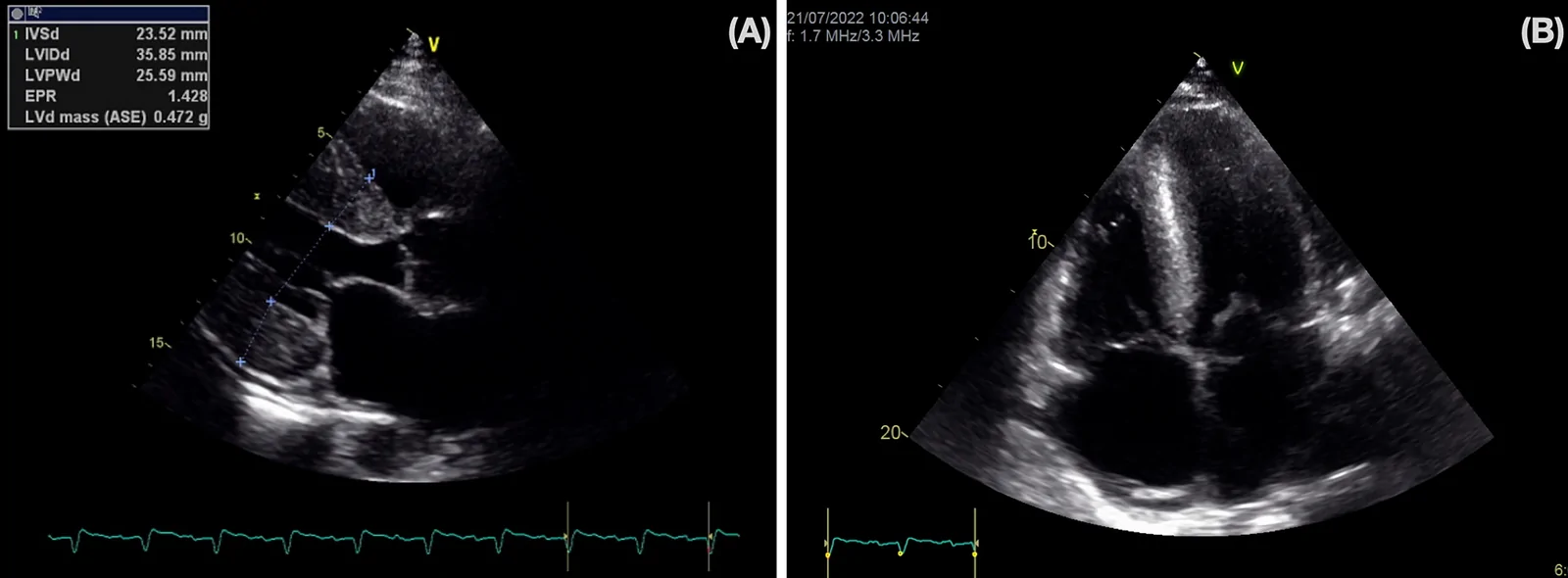
Transthoracic echocardiogram parasternal long axis (**A**) and apical four chamber view (**B**), revealing moderate concentric left ventricular hypertrophy with preserved biventricular systolic function

**Fig. 3:**
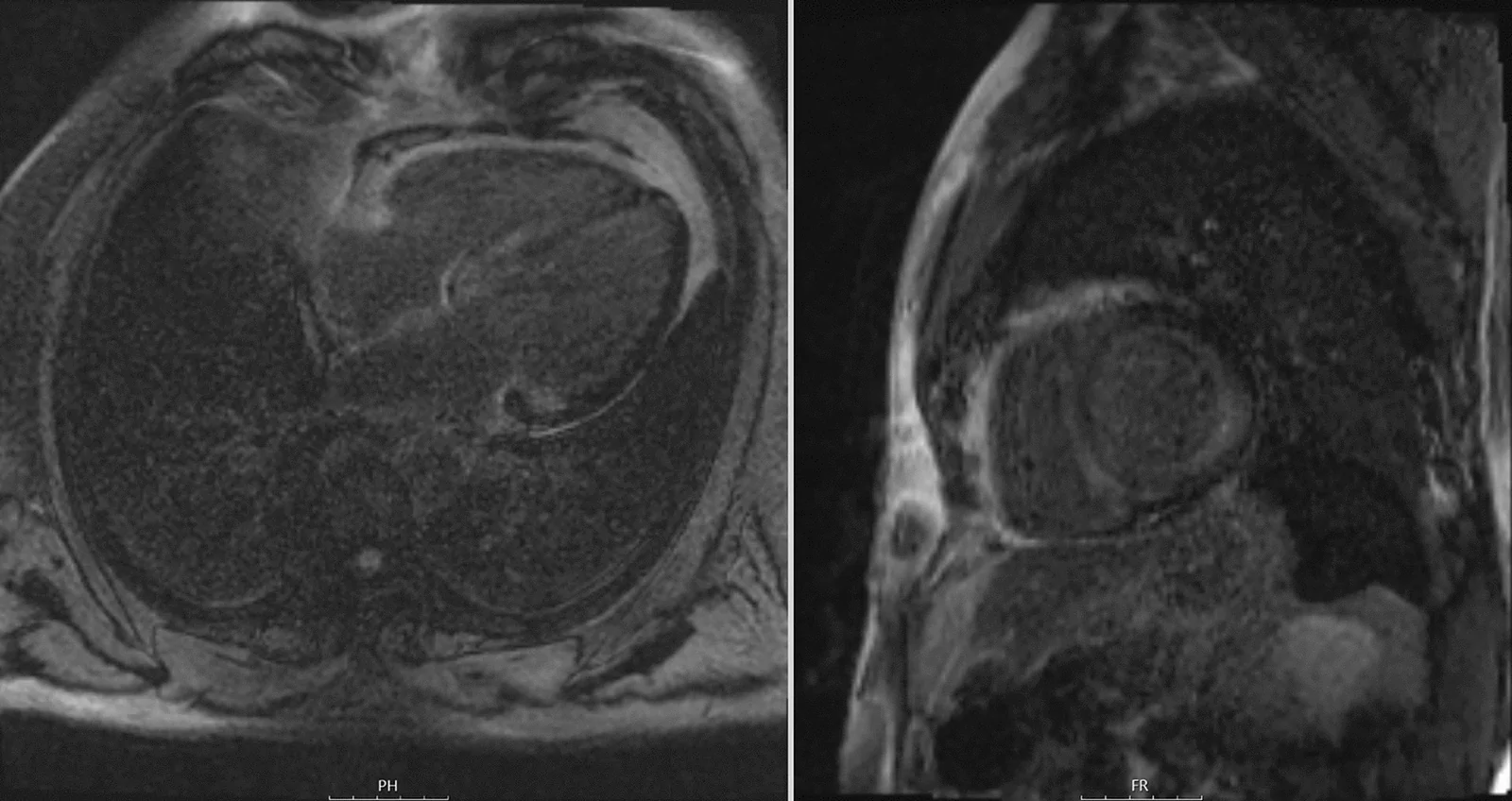
Cardiac magnetic resonance imaging: severe left ventricular hypertrophy (20 mm at the inferoseptal wall); dIffuse left ventricular fibrosis was seen, predominantly in the subendocardium, and more noticeable in the basal segments; late-gadolinium enhancement pattern suggesting infiltrative cardiomyopathy

**Fig. 4:**
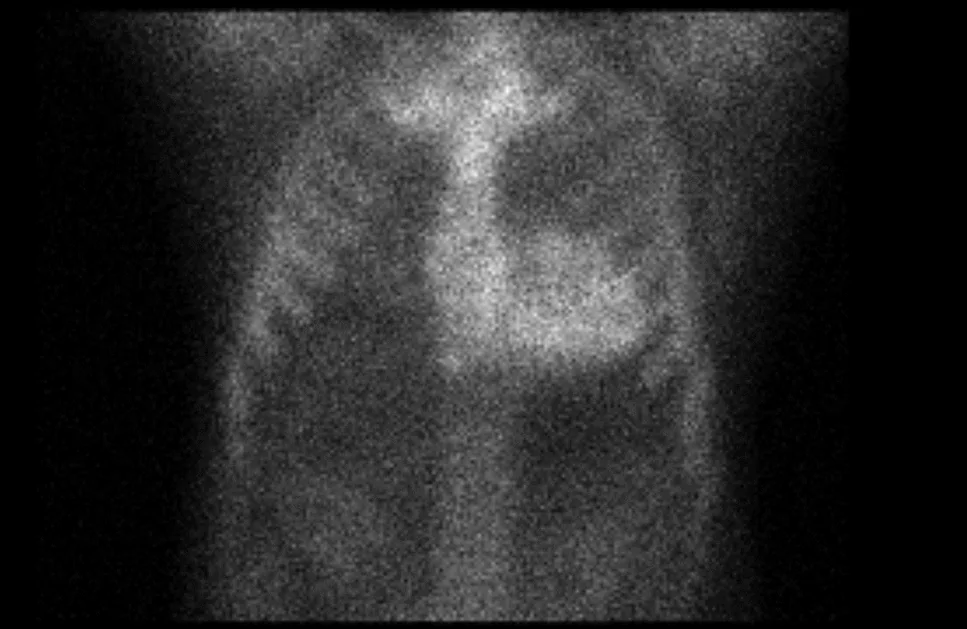
99mTc-3,3-diphosphono-1,2-propanodicarboxylic acid scintigraphy was conducted, showing a cardiac uptake compatible with a Perugini score of 2

A 52-year-old man with no cardiovascular risk factors or known family history of heart disease was referred to a Cardiology Consultation as an outpatient due to complaints of effort-related oppressive chest pain, progressively worsening dyspnea and peripheral edema over the past months. He denied urinary symptoms, namely decreased urinary output or foamy urine, and neurological disorders, namely paresthesias or decreased sensitivity. There was no history suggestive of carpal tunnel syndrome. Electrocardiogram (ECG; Fig. 1) showed sinus rhythm, first-degree atrioventricular block, Q-waves in V1-V3 precordial leads, and T-wave inversion in the inferior and lateral leads. Transthoracic echocardiogram (TTE; Fig. 2) revealed moderate concentric left ventricular hypertrophy with preserved biventricular systolic function. Cardiac magnetic resonance imaging (CMR; Fig. 3) disclosed severe left ventricular hypertrophy (20 mm at the inferoseptal wall). Diffuse left ventricular fibrosis was seen, predominantly in the subendocardium, and more noticeable in the basal segments. Late-gadolinium enhancement (LGE) pattern suggested infiltrative cardiomyopathy. There were no signs of myocardial ischemia. He had normal hemoglobin and renal function; BNP > 14,400 pg/mL; no monoclonal peaks in the protein electrophoresis (Table 1); hypogammaglobulinemia; serum light chain count of lambda/kappa was 0.83 and 30.6 mg/L respectively, with a ratio > 35 (Table 2); urinary light chain count of lambda/kappa was < 0.39 and 12.50 mg/L respectively, with a ratio 32 (Table 3); B2-microglobulin was normal; lactate dehydrogenase (LDH) was only mildly elevated. Genetic screening excluded TTR gene mutations. Dried blood spot testing for Fabry's disease was negative. A ^99m^Tc-3,3-diphosphono-1,2-propanodicarboxylic acid (^99m^Tc-DPD; Fig. 4) scintigraphy was conducted, showing a cardiac uptake compatible with a *Perugini score* of 2. Multiple abdominal fat and salivary gland biopsies were performed with negative results for amyloid. Bone marrow biopsy revealed < 1% plasma cells (with > 99% monoclonality), consistent with monoclonal gammopathy of undetermined significance (MGUS), rather than multiple myeloma (MM) or smoldering myeloma (SM). An endomyocardial biopsy (EMB) was performed, and immunohistochemical staining revealed deposits of kappa light chains compatible with the clinical suspicion of AL. The patient was started on aggressive chemotherapy based on bortezomib and high doses of glucocorticoids. Unfortunately, his condition rapidly deteriorated. He was hospitalized twice in less than a month due to decompensated heart failure. On his last admission, the patient collapsed due to asystole.

**Table 1:** Protein electrophoresis

Type	Level	Reference Range
Total Protein	5.6 g/dL	
Albumin	67.4%	55.8–66.1
Alpha 1 Globulin	5.0%	2.9–4.9
Alpha 2 Globulin	10.4%	7.1–11.8
Beta 1 Globulin	5.7%	4.7–7.2
Beta 2 Globulin	3.6%	3.2–6.5
Gamma Globulin	7.9%	11.1–18.8
IgA	65 mg/dL	114–457
IgG	499 mg/dL	793–1590
IgM	21.1 mg/dL	29–226

**Table 2:** Serum free light chains

Type	Level	Reference Range
Lambda light chain	0.83 mg/dL	< 0.7 mg/dL
Kappa light chain	30.6 mg/dL	< 0.9 mg/dL
K/L ratio	36.957	0.7—6.2

**Table 3:** Urinary light chains

Type	Level	Reference Range
Lambda light chain	< 0.39 mg/dL	< 0.85 mg/dL
Kappa light chain	12.50 mg/dL	< 0.47 mg/dL
K/L ratio	32.05	0.75—4.50

## Discussion

Amyloidosis is a group of systemic diseases caused by the abnormal deposition of insoluble misfolded beta-pleated proteins in various tissues and organs, locally or systemically, leading to organ dysfunction. More than 30 different proteins are known to be involved. CA is a serious and often underdiagnosed complication of amyloidosis. Diagnosis can be challenging, but early diagnosis and management may improve the outcomes. CA is the result of amyloid deposits in the heart, leading to heart failure and other cardiac symptoms. The two most common types of amyloidosis associated with CA are light chain amyloidosis (AL) and transthyretin amyloidosis (ATTR). Accurate diagnosis of the type of amyloidosis is critical for guiding appropriate management and predicting prognosis [1].

AL is caused by the deposition of monoclonal light chains produced by clonal plasma cells. It is also known as primary amyloidosis, and is often associated with plasma cell dyscrasia, such as MM [2]. The prevalence of AL amyloidosis in patients with MGUS and indolent myeloma is significantly lower than in MM. Studies have shown that the incidence of AL in patients with MGUS is approximately 1%, while in patients with MM, the incidence ranges from 5–15% [3]. Similarly, the incidence of AL in patients with indolent myeloma is lower than in MM, but higher than in MGUS [4].

Cardiac involvement is common in AL, with up to 50% of patients showing evidence of cardiac amyloidosis at diagnosis. The prognosis of AL with cardiac involvement is poor, with a median survival of approximately 6 months without treatment [5].

On the other hand, ATTR is caused by the deposition of transthyretin protein, which is normally produced in the liver and circulates in the peripheral blood. ATTR can be further classified into hereditary (hATTR) and wild-type (wtATTR) forms. hATTR is an autosomal dominant inherited disease that is caused by mutations in the transthyretin gene, while wtATTR is sporadic and typically affects older patients. Cardiac involvement is more common in hATTR amyloidosis, with up to 80% of patients showing evidence of cardiac amyloidosis at diagnosis. The prognosis of ATTR patients with cardiac involvement is also poor, but it is typically better than in AL, with a median survival of approximately 2–3 years [6].

A multimodality approach is essential in the diagnosis of CA, combining information from clinical evaluation, imaging, and laboratory data.

Endomyocardial biopsy (EMB) remains the gold standard for diagnosing cardiac amyloidosis, though its limitations include invasiveness and potential sampling error. Therefore, other diagnostic methods have been investigated and are gaining importance in the diagnosis of cardiac amyloidosis.

CMR has emerged as a noninvasive imaging modality that can provide important diagnostic and prognostic information in patients with suspected CA. CMR can identify characteristic patterns of cardiac involvement, such as myocardial thickening, LGE, and T1 and T2 mapping abnormalities. Several studies have shown that CMR can accurately diagnose cardiac amyloidosis, with high sensitivity and specificity, and can provide important prognostic information [7, 8].

Nuclear imaging techniques, including 99mTc-DPD scintigraphy and fluorine-18 fluorodeoxyglucose (F-18 FDG) positron emission tomography (PET), have shown promise in diagnosing cardiac amyloidosis. ^99m^Tc-DPD scintigraphy is a well-established imaging technique for the detection of ATTR and has been shown to have high sensitivity and specificity for the diagnosis of cardiac involvement [9]. F-18 FDG PET has been used in the diagnosis of AL, and can detect amyloid [10].

Serum and urine protein electrophoresis, as well as serum free light chain assays, are also important diagnostic tools, as they can detect the presence of monoclonal immunoglobulin light chains, which are a hallmark of AL [11].

Nevertheless, only EMB allows for the identification of the type of amyloid protein, which is fundamental, as different types of amyloids have different management and prognosis.

The therapeutic approach in cardiac amyloidosis depends on the type of amyloidosis involved. For AL amyloidosis, treatment involves chemotherapy to target the underlying plasma cell disorder, while in ATTR amyloidosis, treatment aims to stabilize or slow the progression of the disease by targeting the abnormal transthyretin protein. This can be achieved through medications, such as tafamidis and diflunisal, that stabilize the transthyretin tetramer, or through liver transplantation, which can reduce the production of the abnormal protein [12].

MGUS without cardiac involvement typically does not require treatment, and patients are usually monitored for disease progression. The risk of progression to MM or other lymphoproliferative disorders in MGUS without cardiac involvement is low, with an annual risk of approximately 1% [13]. However, in patients with MGUS-associated CA, the prognosis is worse, and the therapeutic approach is focused on managing the underlying systemic amyloidosis, with the goal of preventing or delaying progression of the disease and improving quality of life.

The development of new treatments, including immunotherapy and gene therapy, may offer hope for improving the management and outcomes in patients with AL. However, to identify patients who will most benefit from each treatment, histological diagnosis is essential. The primary goal of treatment in AL amyloidosis is to reduce the production of amyloidogenic light chains by controlling the underlying plasma cell disorder. The choice of chemotherapy regimen depends on patient age, comorbidities, and the extent of organ involvement. Commonly used chemotherapy regimens include bortezomib and dexamethasone, cyclophosphamide, bortezomib, and dexamethasone, and melphalan and dexamethasone. In patients with severe renal involvement, plasma exchange or dialysis may be required to reduce the concentration of circulating light chains [14–16].

Early diagnosis and prompt treatment with ‘source therapies’ that limit the production of amyloidogenic LC are associated with better survival and improvement in organ function after a median of 2.4 months following haematological complete response. However, organ recovery is often incomplete because these source therapies do not directly target deposited amyloid. Emerging amyloid- directed therapies may attenuate, and potentially reverse, organ dysfunction by clearing existing amyloid and inhibiting fibril formation of circulating aggregates. Improved recognition of AL amyloidosis by cardiologists allows for earlier treatment and improved outcomes.

## Conclusion

This case highlights the diagnostic challenges and underscores the crucial role of EMB in selected cases. Despite age and rapid deterioration favoring AL amyloidosis, non-invasive tests were inconclusive. Persistent clinical suspicion led to invasive methods, aiding a final albeit delayed diagnosis.

## Data Availability

The authors confirm that the data supporting the findings of this study are available within the article.
